# Mitochondrial DNA replication: a PrimPol perspective

**DOI:** 10.1042/BST20160162

**Published:** 2017-04-13

**Authors:** Laura J. Bailey, Aidan J. Doherty

**Affiliations:** Genome Damage and Stability Centre, School of Life Sciences, University of Sussex, Brighton BN1 9RQ, U.K.

**Keywords:** DNA replication and recombination, mitochondria, polymerase, primase, PrimPol, repriming

## Abstract

PrimPol, (primase–polymerase), the most recently identified eukaryotic polymerase, has roles in both nuclear and mitochondrial DNA maintenance. PrimPol is capable of acting as a DNA polymerase, with the ability to extend primers and also bypass a variety of oxidative and photolesions. In addition, PrimPol also functions as a primase, catalysing the preferential formation of DNA primers in a zinc finger-dependent manner. Although PrimPol's catalytic activities have been uncovered *in vitro*, we still know little about how and why it is targeted to the mitochondrion and what its key roles are in the maintenance of this multicopy DNA molecule. Unlike nuclear DNA, the mammalian mitochondrial genome is circular and the organelle has many unique proteins essential for its maintenance, presenting a differing environment within which PrimPol must function. Here, we discuss what is currently known about the mechanisms of DNA replication in the mitochondrion, the proteins that carry out these processes and how PrimPol is likely to be involved in assisting this vital cellular process.

## Mitochondrial DNA — organisation and structure

Mammalian mitochondria contain multiple copies (∼1000 per cell) of a circular DNA molecule (mtDNA) that is ∼16.5 kb in length [1]. Unlike nuclear genomic DNA, virtually the entire mtDNA encodes genes that are expressed as 13 proteins, 22 tRNAs and 2 rRNAs with no introns. Only two non-coding regions exist, the non-coding region (NCR), containing the origin of heavy-strand DNA replication and the transcription initiation start sites HSP and LSP (light- and heavy-strand promoters), and O_L_, the origin of light-strand DNA replication.

**Figure 1.**
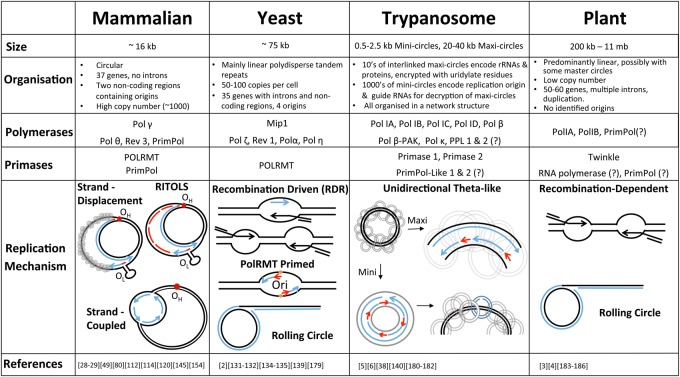
The diversity of mitochondrial genomes. This table presents the wide variety of mtDNA sizes found across different kingdoms and how their organisation and replication mechanisms differ. Also highlighted are primases and polymerases shown to be, or speculated to be (?), involved in these processes. Black strands represent parental DNA, with newly synthesised DNA shown in blue and RNA in red.

Strikingly, significant physical differences exist between the mitochondrial genomes of different eukaryotic organisms (Figure 1). For example, most yeast species have linear mitochondrial genomes ranging in size from ∼19 to 150 kb, often consisting of circular permutated copies [2]. In plants, the mitochondrial genome varies even more in size, from ∼200 kb up to a massive 11 Mb, due to a large number of introns and duplications (reviewed in refs [3,4]). In contrast, kinetoplast DNA found in the mitochondria of some protists (e.g. *Trypanosoma*) contains two types of DNA circles, large maxi-circles (20–40 kb) containing the majority of the coding DNA that are catenated with smaller mini-circles (0.5–10 kb), which are essential for the production of functional mRNA from the uridylate encrypted maxi-circles [5,6].

In mammalian cells, mtDNA is organised into nucleoid structures containing ∼1–2 copies of the genome, although this is likely to vary depending on tissue type and energy demand [1,7,8]. Mitochondrial nucleoids are thought to tether mtDNA to the inner mitochondrial membrane to aid in organisation, distribution and segregation. Nucleoids consist of many proteins involved in the maintenance of mtDNA, but the main DNA packaging protein is TFAM, mitochondrial transcription factor A [9,10]. TFAM is a member of the high-mobility group (HMG) of proteins, containing two HMG boxes, and is thought to condense DNA by inducing bending and then wrapping it into compacted nucleoid structures. TFAM forms homodimers with each molecule binding a separate DNA strand in order to generate looped structures or to bring different DNA molecules together leading to condensation (reviewed in ref. [11]).

One striking feature of mammalian mtDNA is a short triple-stranded region found within the NCR, where the addition of a third DNA strand (∼0.5 kb), termed 7S DNA, forms a stable displacement D-loop structure [12]. The D-loop was first identified in electron micrograph images of mtDNA and the 7S DNA was later found and named due to its sedimentation rate in caesium chloride gradient studies [12,13]. Despite the length of time since the first identification of 7S DNA and the D-loop, we still know little about its actual function (reviewed in ref. [14]). Its abundance is found to vary greatly between species and tissues, with only 8% of mtDNA molecules having D-loops in HeLa cells, while levels of 7S DNA are as high as 55% in human placenta [15]. The D-loop is thought to have many roles, including acting as a recruitment site for proteins involved in the organisation of mtDNA into nucleoid structures [16,17], maintaining dNTP pools throughout the cell cycle [18] and functioning as a key component of replication (see below). Recent work has shown that this structure may actually be more complex with the identification of an RNA strand on the opposing strand to the 7S DNA forming an R-loop, which may have a role in the organisation and segregation of mtDNA [19].

A wide range of proteins are involved in the organisation, regulation and replication of the mitochondrial genome. All of these proteins are encoded within the nuclear genome and must be transported into the organelle as required. Initially, only a few specialised proteins were thought to be required for these processes but, as techniques have improved, we are now identifying numerous additional factors that play roles in the maintenance of mtDNA. Many of these proteins function in both nuclear and mitochondrial compartments, such as DNA2, Fen1, PIF1, Rad51C and XRCC3; some require specialised isoforms, such as Fen1 and PIF1, whereas others use the same isoform to perform these dual roles [20–26].

## PrimPol — a novel eukaryotic primase–polymerase

A recent example of such a player is primase–polymerase (PrimPol), a newly discovered member of the archaeo-eukaryotic primase (AEP) superfamily of primases [27–30] (reviewed in refs [31,32]). As its name suggests, PrimPol can function both as a primase and a polymerase. Many studies have demonstrated that PrimPol is capable of generating both DNA and RNA primers on single-stranded (ss) DNA templates, albeit with a strong preference for dNTPs during synthesis, ∼30-fold lower *K*_d_ in Mn^2+^ [28,29,33]. In addition, PrimPol is also capable of extending a DNA primer in a template-dependent manner and is capable of carrying out translesion synthesis (TLS) across many different DNA lesions, such as 8-oxoguanine (8-oxoG) and 6-4 photoproducts (6-4 PP) [28,29]. PrimPol contains an N-terminal AEP domain, which contains the key catalytic residues required for all its synthesis activities. Its C-terminal UL52-like zinc finger domain is essential for its priming activity, probably by stabilising the protein's interaction with ssDNA to allow *de novo* dinucleotide synthesis that is subsequently extended to form a primer [34]. PrimPol is a remarkably unprocessive polymerase, catalysing the insertion of only 1–4 nucleotides before disassociating from DNA, and is highly mutagenic, particularly prone to produce insertion/deletion errors [34–36]. The limited processivity of PrimPol may act to limit its mutagenic potential. The elucidation of the structure of the catalytic core of PrimPol has highlighted its relatively small active-site cleft, with limited contacts formed between the protein and the incoming primer strand [37].

PrimPol is localised in both the nucleus and the mitochondrion, suggesting that it plays similar roles in maintaining DNA integrity in both compartments [28,29]. Notably, avian cells (DT40) lacking PrimPol exhibit increased sensitivity to many DNA-damaging agents and exhibit a pronounced G_2_ arrest after exposure to Ultraviolet (UV) damage [28,29]. Loss of a PrimPol orthologue (PPL2) in *Trypanosoma* is lethal due to a failure to complete cell division in G_2_/M phase [38]_._ In contrast, loss of PrimPol alone is not overtly detrimental to mammalian cells, with no obvious signs of damage sensitivity, while knockout mice are viable and born at Mendelian ratios [28,29]. These differences probably reflect the replication poise of these cells as DT40 cells are mostly in S-phase, while mammalian cells sit predominantly in G_1_ phase. However, human cells become significantly more sensitive to UV damage when PrimPol and Pol η, a damage tolerance TLS polymerase, are both absent [28,39,40]. Strikingly, loss of PrimPol causes an increase in mtDNA copy number and cells exhibit reduced rates of mtDNA recovery after ethidium bromide-induced mtDNA loss, suggesting that it is important for maintaining ‘genome’ stability within this organelle [28,29,41]. PrimPol has also been shown to functionally interact with many other proteins from both nuclear and mitochondrial compartments. These include mitochondrial single-stranded DNA-binding protein (mtSSB), replication protein A (RPA), polymerase delta interacting protein 2 (PolDIP2) and Twinkle, the mitochondrial helicase, which all appear to play roles in regulating PrimPol's cellular activities [35,42,43].

However, much is still to be learned about the function of PrimPol in the maintenance of replication in both mitochondrial and nuclear compartments. Here, we review what is currently known about DNA replication processes in the mitochondrion and discuss how our newfound knowledge of PrimPol's activities informs us about its possible roles in the duplication of mtDNA.

## Priming mtDNA replication — how it all begins

### The elusive mitochondrial primase

To begin replication, DNA must first be ‘primed’ by the generation of short primers, which the replicase is able to extend. In the nucleus, RNA primers are synthesised on ssDNA by the Pol α-associated primase (PriS/Pri1-DNA Primase small subunit) (reviewed in refs [44,45]). However, the enzyme responsible for the initiation of mammalian mtDNA replication has taken much longer to be discovered and we are only now beginning to unravel how this process occurs. A mitochondrial primase activity was first identified back in 1985 [46]. This primase activity isolated from mitochondria was distinct from that of the replicative polymerase γ (Pol γ) and the mitochondrial RNA polymerase (POLRMT), and was shown to have the ability to catalyse the formation of RNA primers 9–12 nucleotides long; however, the enzyme responsible for this activity was not identified [46]. This activity was further characterised to show that the primase was capable of generating a 5′ oligoribonucleotide primer with a 3′ deoxyribonucleotide termini for polymerase extension and was associated with a structural RNA molecule, which is essential for its activity [47,48]. Although some further insights were gained in other species and models of mtDNA replication were proposed, little more was learnt about DNA priming or the elusive ‘primase’ for the next 20 years. The subject of DNA replication initiation in the mitochondrion was reignited with the discovery that the mitochondrial RNA polymerase, POLRMT, was capable of initiating lagging-strand DNA replication *in vitro* [49].

### POLRMT — a mitochondrial primase

Mitochondrial RNA polymerase, like the majority of mitochondrial proteins, is encoded within the nuclear genome and contains an N-terminal mitochondrial targeting sequence, which localises it to this organelle. POLRMT is a single-subunit RNA polymerase, which shares significant homology with phage RNA polymerases [50,51]. The C-terminal domain contains the catalytic core and consists of many conserved sequence blocks, which form the characteristic thumb and palm structure. Unlike the C-terminus, the N-terminal portion of the protein has no sequence similarity to the phage polymerases; instead, this region is structurally related to the T7 RNA polymerase (reviewed in ref. [52]). This structural homology is also mirrored in its functional uses as phage T7 replication employs the RNA polymerase in a similar manner to generate RNA primers [53–55]. In addition, POLRMT contains a unique N-terminal extension, containing a novel pentapeptide repeat domain that is essential for promoter-dependent transcription [56]. Unlike T7 RNA polymerase, POLRMT is unable to bind to, bend and melt promoter DNA alone and requires many accessory factors to begin transcription. These factors include TFAM, which is thought to alter the DNA structure, allowing initiation, and TFB2M, which interacts directly with the RNA polymerase to aid its recruitment [57–61]. Similarly, yeast mitochondrial RNA polymerase Rpo41 requires transcription factor Mtf1 for tight DNA binding (reviewed in ref. [62]). POLRMT must be targeted to a range of diverse promoter sequences and additional factors are likely to be important in its localisation to different structural DNA elements (e.g. O_L_). The generation of POLRMT conditional knockout mice, which have no identifiable 7S DNA, confirmed that POLRMT has an essential role in replication initiation [63].

Mitochondrial RNA primers (2–10 nucleotides) were first identified at the 5′ end of the 7S DNA back in 1979 [64]. More recent work has identified that these primers are generated by POLRMT [49,65,66], originating from the LSP and HSP, within the NCR [67,68]. As well as acting as the start site for transcription of the mitochondrial genome, LSP was shown to also be the initiation site for the formation of a persistent RNA–DNA hybrid, believed to act as a primer for leading-strand DNA replication [69]. Transcription initiated from the LSP suffers two fates: it is either terminated within a region containing three conserved sequence blocks (CSBs) and used as a primer for mtDNA replication, or extension continues around the mtDNA molecule to form a polycistronic transcript, which is then processed further [70,71]. The ends of this short RNA were shown to map close to previously identified sites of DNA replication initiation, and this RNA species was found to be sufficient for the initiation of replication *in vitro* [69,72].

Later work showed that CSB II, a conserved sequence box within the NCR, acted as the termination site for transcription, controlling the formation of a primer rather than extension to complete transcription [66]. CSB II has the potential to form a quadruplex structure, due to its G-rich sequence. However, although this region can form a DNA quadruplex, the majority of these species were actually found to form RNA–DNA hybrid intermolecular quadruplexes [73,74]. These structures act as strong transcription terminators, leading to the formation of primers. Thus, the CSB II quadruplex acts as a switch regulating the interplay between transcription and replication of mtDNA. The analysis of variation in the CSB II region of human mtDNA has shown that sequences that cause only weak termination of POLRMT and transcription are avoided. However, all transcription termination events are localised to the same downstream sequences, revealing the importance of the conservation of this region [75]. Yet, it is still important that when required, these RNA molecules are extended into full-length species to provide sufficient mRNA, and therefore protein, as required by the organelle. This switch is also partly controlled by human transcription elongation factor, TEFM, shown to prevent replication primer formation by driving transcription elongation [76,77]. However, the polymerase must then extend the primer beyond this quadruplex structure, and it has been shown that Pol γ exhibits a much decreased extension ability on the CSB II sequence, compared with a mutant CSB II sequence that is unable to form a quadruplex structure [73]. In addition, these primer ends fall short of the identified 5′ ends of newly replicated DNA molecules [78,79], suggesting things may not be quite so straightforward; therefore, we still have much more to learn about the mechanisms that initiate priming of mtDNA replication in the mitochondrion.

MtDNA is double-stranded (ds) and, therefore, replication must also be initiated on the other strand. The origin of light-strand replication, O_L_, is located approximately two-thirds of the way around the genome from the NCR and resides in a much smaller NCR of DNA occupying only 30 base pair (bp), between the asparagine and cysteine tRNA genes [80]. POLRMT can generate short RNA primers at O_L_, allowing complete leading- and lagging-strand replication (when combined with Pol γ, Twinkle and mtSSB) of a small circular DNA substrate *in vitro* [49]. Further studies *in vivo* confirmed that POLRMT is capable of priming specifically at O_L_ and demonstrated that this specificity is due to a stem loop structure formed when the O_L_ sequence is exposed after leading-strand replication uncovers this region [81]. This stem loop structure is highly evolutionarily conserved and mutations affecting its structure are significantly under-represented in the mitochondrial genome, while insertions and deletions in the loop region are well tolerated, confirming the importance of such a structure within the mitochondrial genome [82].

### PrimPol — the elusive mitochondrial primase?

The discovery of PrimPol within the mitochondria raises many important questions regarding its role as a primase within mtDNA replication [28,29]. Although PrimPol has the ability to generate both DNA and RNA primers [28,29], it has a strong preference for utilising dNTPs rather than rNTPs to synthesise primers, and studies using PrimPol isolated by fractionation of purified mitochondria showed that the enzyme generated DNA primers of ∼2–12 nt in length [29]. This is in clear contrast with the original activity of the historically unassigned ‘mitochondrial primase’; thus, PrimPol appears not to be this elusive protein and suggests that there is still much more to be uncovered. If it is not the replicative primase, then what are PrimPol's primase activities required for within the mitochondrion? Mammalian PrimPol is a non-essential protein as knockout cell lines and mice strains have been generated, which exhibited no overt phenotypes [28,39]. Thus, it appears that the role of PrimPol can be complemented by other proteins within the organelle or it may itself have a ‘back-up’ role, which may be only required under certain particular circumstances. Therefore, it is clear that PrimPol is not the main enzyme responsible for priming of mtDNA replication and, notably, mtDNA copy number actually increases in the absence of PrimPol [28]. It seems more likely that PrimPol acts to reprime DNA synthesis, or bypass lesions by TLS, when the replicase is stalled or blocked.

## Mitochondrial DNA replication — copying the circle

### Pol γ — the mitochondrial polymerase

Although a wide range of polymerases are required to replicate and repair nuclear DNA, only one polymerase was identified within the mitochondria and this was believed to be responsible for all of the replication and repair processes that take place within this organelle [83]. Although Pol γ was first identified as early as 1977, it took many more years for it to be isolated from *Drosophila* and confirmed as the key mitochondrial replicase due to the abundance of nuclear polymerases [84,85]. Pol γ is a heterotrimeric complex consisting of two nuclear-encoded components. POLG1 or A is a 140 kDa subunit belonging to the PolA family of polymerases and is thought to share a common ancestry with the T-odd polymerase gp5 [86]. It is essential for mtDNA maintenance and its loss is embryonic lethal [87]. As well as 5′–3′ polymerase activity, POLG1 also contains a highly conserved 3′–5′ exonuclease domain, which confers a key part of its high fidelity, ∼100-fold greater than that observed for nuclear polymerases [88,89]. In addition, POLG1 also has 5′ dRP lyase activity, which allows the enzyme to execute base excision repair, again increasing its fidelity and strengthening its role in DNA repair within mitochondria [90]. The crystal structure of the Pol γ complex has provided further insights into its mechanism and the unique interactions between its subunits. The analysis of a wide number of disease-causing mutations within the context of this structure has led to a greater understanding of the molecular changes that these alterations induce [91,92] (reviewed in ref. [93]).

POLG2 or B is a 55 kDa homodimeric accessory subunit required for tight binding of the holoenzyme on DNA [94]. It is also vital for increasing the processivity of the replication complex and interacts with Twinkle at the replication fork [95,96]. POLG2 evolved from class IIa aminoacyl tRNA synthases and appears to be less conserved than its catalytic counterpart, with no orthologues identified in fungi [97]. The binding of POLG2 significantly increases the processivity of the enzyme due to a change in the structure of POLG1, which increases its DNA interaction ‘footprint’ from 10 to 25 bp [91]. The addition of POLG2 to the complex decreases the proofreading capacity of the enzyme, which is probably due to a decrease in the ability to switch the template DNA from the polymerase to the exonuclease active site [88,96].

The polymerase is assisted in the completion of replication by many other components including Twinkle, the mitochondria helicase and MtSSB (SSBP1), a single-stranded DNA-binding protein. More recently, a much wider range of proteins have been associated with mtDNA replication and repair, many of which have already been identified based on their roles in nuclear DNA metabolism. These include the helicases DNA2, PIF1 and RecQL4, topoisomerases TopImt, TopIIIα, TopIIβ [21–24,98–101] and Fen1, DNA ligase III and RNAse H1 [25,102–106].

### Mechanisms of mammalian mtDNA replication

The first studies of mtDNA replication were carried out using electron microscopy, which highlighted many unique structures within the observed molecules, including double-forked structures with one single-stranded branch [107–109]. These studies led to the proposal of a strand-displacement model of mtDNA replication [108,110,111]. This model proposes that replication continues in an asynchronous, unidirectional manner; replication proceeds on the H-strand extending from the primer generated in the NCR, while the unreplicated ssDNA becomes coated with mtSSB. After replication has proceeded two-thirds of the way around the molecule, then O_L_ becomes exposed, allowing replication to be primed in the reverse direction, again continuing unidirectionally around the circle to generate a fully duplicated daughter molecule [112].

The use of 2D neutral agarose gel electrophoresis (2D AGE) uncovered a second distinct replication mechanism of mtDNA replication. This technique relies on the fact that DNA can be isolated dependent on its mass and shape by altering separation conditions [113]. Strikingly, when mtDNA replication intermediates were examined by this method, many conventional replication bubbles and forks were observed. These structures were resistant to ssDNA nucleases, suggesting a conventional strand-coupled mechanism of replication [114–117]. Although such dsDNA molecules had been observed much earlier in electron microscopy (EM) images of rat liver mtDNA, a strand-coupled mechanism was not pursued [108,109,118,119]. Replication is believed to initiate in a bidirectional manner from a broad zone termed *ori Z*; however, O_H_ acts as a strong replication barrier; therefore, DNA replicases are stalled and unable to pass bidirectionally through this region [114,116].

In addition to conventional strand-coupled replication intermediates, 2D AGE also identified replication intermediates with significant regions of RNA–DNA hybridisation [79,117]. This lead to the proposal of the Ribonucleotide Incorporation ThroughOut the Lagging Strand (RITOLS) mechanism of mtDNA replication, where RNA is bound to the displaced strand during asynchronous replication rather than mtSSB [79]. This RNA was identified as mtDNA transcripts, which are thought to be laid down when the L-strand is released as ssDNA during replication and *in organello* labelling allowed these RNA species to be followed as replication proceeds [120]. More recent EM studies support this model as it was observed that when conditions were optimised to preserve fragile RNA, no ss mtDNA regions were present [121]. Variations in the types of replication intermediates were observed across different tissues and under different stresses (e.g. damage or mtDNA depletion), suggesting that different mechanisms may be utilised under various circumstances [122].

However, there is still much controversy and disagreement over these different replication mechanisms, with recent work showing that mtSSB occupancy is increased between O_H_ and O_L_ on the displaced heavy strand, favouring the strand-displacement model [123]. While it has been claimed that mtSSB is lost due to proteinase K treatments used to observe RITOLS intermediates and that R-loops are prevalent in mtDNA but not replication intermediates, others claim that RNA loss during preparation leads to mtSSB binding [121,123,124]. Although more studies are required to fully unravel the mechanisms of mtDNA replication, it seems likely that this process consists of a combination of the proposed models.

Although it currently seems unlikely that PrimPol plays a direct role in replicating mitochondrial DNA, as it is non-essential in mammalian cells, it probably plays roles in maintaining ongoing replication when the replicative complex of Pol γ and Twinkle hits an obstacle. Cells are clearly capable of maintaining replication in the absence of PrimPol and, notably, the majority of yeast species lack PrimPol orthologues. However, the presence of this additional primase–polymerase is likely to aid in overcoming replication barriers, thus ensuring timely completion of replication.

### Damage tolerance during mtDNA replication

For many years, it was believed that mitochondria did not have their own DNA repair proteins/pathways as they were unable to remove cyclobutane pyrimidine dimer (CPD)s after UV damage [125]. Thus, replication must be able to proceed beyond such damage, which is likely to be perpetuated within the DNA. However, these original ideas have now been refuted and many new players in the maintenance and repair of mtDNA are regularly being added to the team of proteins maintaining the ‘genome’ within this organelle. Although a wide range of repair pathways are now known to be able to remove DNA lesions within mitochondria, this can be a relatively slow process and, therefore, Pol γ probably requires additional damage tolerance/bypass mechanisms to allow timely progression of replication.

### Bypass of lesions — overcoming obstacles on DNA

**Figure 2.**
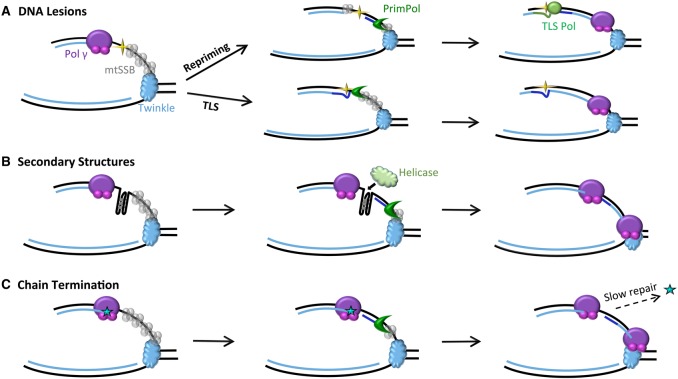
Potential functions of PrimPol during mtDNA replication. Highlighted here are ways in which PrimPol may play key roles in allowing the maintenance of mtDNA replication in many fork-stalling situations. (**A**) After Pol γ is stalled by a lesion (yellow star), PrimPol is capable of repriming synthesis downstream to allow replication to proceed while the slower process of replication across the lesion is dealt with by Pol γ itself or another specialised TLS polymerase. Alternatively, PrimPol may act as a TLS polymerase that directly bypasses the lesion. (**B**) PrimPol may play a role in repriming synthesis when the replication fork is stalled by DNA secondary structures, e.g. G4s. By priming after the structure, it allows replication to continue downstream, while specialised helicases are recruited to facilitate synthesis through the structure. (**C**) Nucleoside analogues (cyan star), incorporated into the newly synthesised strand, prevent further elongation and must be removed by the exonuclease of Pol γ, which is a slow process. PrimPol may reprime downstream from CTNAs to allow replication to continue in a timely fashion, while the process of removing these is completed. In each case, the second DNA strand is shown as dsDNA for simplicity; however, this could be coated with mtSSB or RNA transcripts, depending on the mode of replication.

Along with its ability to generate primers, PrimPol is also a template-dependent polymerase with the ability to bypass some lesions. For example, it has the ability to perform TLS bypass of 8-oxoG lesions, a prevalent product of oxidative damage but is unable to bypass abasic sites or thymidine glycol lesions unless supplemented with manganese [28,29]. In addition, PrimPol has the ability to bypass damage caused by UV exposure and, unlike known mammalian polymerases, PrimPol can bypass distorting 6-4 PP lesions that induce DNA bending. Although it is not able to directly bypass CPDs in the presence of magnesium, it can extend from this lesion when templated with two dA residues, unlike many other polymerases [28]. In addition, PrimPol has been reported to scrunch the template, realigning the priming strand in order to bypass intolerable DNA lesions, which seems the more likely mode of bypass given the protein's small active-site cleft [36,37]. Thus, it seems likely that PrimPol may also play a role in the TLS bypass of these types of damage within the mitochondrial genome (Figure 2).

UV damage has been shown to accumulate in mitochondrial DNA, particularly in skin cells that are regularly exposed to low levels of UV irradiation (reviewed in ref. [126]). These lesions are commonly repaired by the nucleotide excision repair pathway [127]. However, this pathway has not been identified within the mitochondria; therefore, such lesions must be repaired by other pathways such as base excision repair (BER), mismatch repair (MMR), non homologous end-joining (NHEJ) or a homologous recombination and may even persist within the DNA. To prevent the catastrophic formation of double-strand breaks during DNA replication, the organelle must have a method to bypass such lesions. Studies have shown that Pol γ has the ability to bypass CPD lesions *in vitro*, albeit at a level much lower than that of undamaged DNA [128]. Although it often incorporates the incorrect base at such sites, it is still able to excise these mutations using its exonuclease domain. However, such methods of bypass either by PrimPol or Pol γ are likely to significantly delay the completion of replication. Although the mitochondria have multiple DNA copies, unperturbed replication of the full genome is thought to take up to 1 h [110]; therefore, only a small amount of damage is likely to have a drastic effect on the organelle's functionality as a whole. Indeed, although low levels of UV or oxidative damage do not affect the mitochondrial DNA copy number, they do cause changes in the mechanism of DNA replication [129]. It has been shown that after such UV damage, replication switches from a RITOLS mechanism to a strand-coupled mechanism, probably due to its ability to complete replication more rapidly and allow the mtDNA copy number to be maintained in the presence of damage [129]. It is believed that the RITOLS mechanism may represent a high-fidelity method of genome replication, while the strand-coupled mechanism may be more error-prone but allow replication to be completed. Notably, tissues with a higher oxidative stress load use a higher degree of strand-coupled replication [122,129]. However, for strand-coupled replication to be successful, particularly in the presence of DNA damage, the replication machinery must be able to generate multiple new primers to allow the continuation of the replication fork.

PrimPol can also reprime DNA synthesis after lesions and it is this function that is likely to be vital in allowing replication to proceed after DNA stalling. Allowing the replication fork to continue beyond such lesions would significantly decrease the completion time and thus allow processes that take a longer time, such as repair of the lesion itself, to take place in a post-replicative fashion. PrimPol is a poorly processive polymerase, only capable of incorporating 1–4 bases before falling off the DNA, suggesting it may be required to produce short primers for the more accurate and exonuclease-containing polymerase, Pol γ, to take over and complete mtDNA replication. PrimPol interacts with RPA, mtSSB and PolDIP2, which modulate its synthesis activities *in vitro* [35,42]. Although PrimPol's polymerase activity is stimulated by Twinkle [43], mtDNA replication studies showed that PrimPol is unable to enhance replication after Pol γ stalling at oxidative lesions [43]. Therefore, although it has the capability to perform TLS *in vitro*, the jury is still out on whether or not PrimPol actually uses such a mechanism to restart replication *in vivo*.

In addition, POLRMT may also play a key role in repriming DNA replication within the mitochondrion. Although POLRMT has been demonstrated to initiate replication at three defined sites in the genome, it is also capable of generating RNA primers on ssDNA [49,130,131]. In contrast with PrimPol, it generates RNA primers of ∼25–75 nucleotides long [49,130], although these have been found to be as short as 9–18 nt in yeast in the presence of the transcription factor Mtf1 [131]. These differences suggest that both may play key roles in repriming but under different circumstances.

### More than one polymerase on the block

Unlike mammalian mitochondria, which for many years were thought to contain only a single DNA polymerase, many additional polymerases have been found to also reside within yeast mitochondria. Like mammalian mtDNA replication, the main replicative polymerase is a Pol γ homologue, Mip1, that consists only of the large catalytic subunit, with no PolG2 homologue identified [132,133]. In addition, Pol α has also been identified within the mitochondria in *Saccharomyces cerevisiae* [134]. Its dual localisation was confirmed by immunofluorescent labelling and although its function within the organelle is yet to be elucidated, it is likely to have a role in repair and gap-filling as it is unable to rescue cells lacking Mip1 [134]. In another *S. cerevisiae* study, Pol ζ, consisting of Rev3, Rev7 and Rev1, was also found to localise within the mitochondria and in the nucleus [135]. While depletion of Rev3 or Rev7 had no effect on mitochondrial mutation levels, loss of Rev1 led to a decrease in the frequency of spontaneous mutations within the mtDNA [135]. Further studies have shown that, although loss of Pol ζ causes a decrease in spontaneous and UV-induced frame-shift mutations, it also causes a large increase in point mutations after UV-C damage, suggesting that an alternative, more mutagenic, pathway is used in its absence [136]. Interestingly, overexpression of Pol ζ is capable of rescuing pathological Mip1 mutants, which cause an increase in mutations [137], while overexpression of the protein in the nucleus causes an increase in mutations [138]. In addition, a proportion of Pol η has also been identified within the mitochondria of budding yeast [139]. Like Pol ζ, it was also found that loss of Pol η causes an increase in the number of mutations produced after UV-C damage within the mitochondrial DNA, confirming an important role in the maintenance of DNA integrity. Other organisms have also been found to have a much wider range of mtDNA polymerases (Figure 1), with trypanosomes having a large number of Pol β- and Pol κ-like polymerases [6,140].

### New players join the mammalian mitochondria polymerase team

A recent study using an mtDNA-specific damaging agent has provided clear evidence for additional polymerases within mammalian mitochondria. Pol θ is a member of the A family of DNA polymerases but, unlike many of the other members, it is a highly promiscuous polymerase that exhibits a wide range of activities on a broad variety of substrates. Indeed, it appears to have roles in interstrand cross-link repair, BER, microhomology-mediated end-joining, TLS, as well as having lyase activity [141–143] (reviewed in ref. [144]). Although it has no mitochondrial targeting sequence, it is recruited to the organelle after oxidative damage [145]. Strikingly, loss of Pol θ leads to a decrease in cellular oxygen consumption and mitochondrial membrane potential, indicative of decreased oxidative phosphorylation, while mtDNA actually shows a decrease in mutations, suggesting that other types of damage may abound [145]. This is not unique, as loss of Pol ζ in the nucleus has a similar effect on mutation levels [146,147]. Although these TLS polymerases are useful for maintaining replication across lesions, thus preventing the formation of possible DSBs (double strand break) as replication and the cell cycle progresses, their ability to perform TLS comes with a high mutagenic cost. Therefore, the cell must balance such consequences to ensure that a functional copy of the genome is passed on to daughter cells. In the mitochondrion, mutations represent less of a risk than in the nucleus, as each gene is available in multiple copies, thus any mutated copy represents a much smaller fraction of the available product pool. Although mutations have been shown to cause a range of mitochondrial diseases, some with catastrophic consequences for cellular survival, these mutations must cross a threshold level before their effects are observed. The mitochondrial DNA copy number appears to be relatively flexible and it has been reported, in many model organisms, that copy number varies widely across tissues and with age [148,149]. Interestingly, the mtDNA copy number has been found to vary widely across different cancers, probably due to mutations in many regulatory genes and, in many cases, mtDNA copy number has been linked to prognostic outcomes [150–152]. In addition, it has been reported that high levels of random mutations are tolerated by cells. For example, studies on mice lacking the exonuclease domain of Pol γ showed premature ageing. However, when mutation levels were analysed in the heterozygotes, they were shown to be almost as high as homozygote litter mates but showed no premature ageing phenotypes [153].

In addition, extension of work initially carried out in yeast has shown that a specific isoform of Rev3 is also found within mammalian mitochondria, yet unlike in yeast, the other components that form Pol ζ have not been identified [154]. Loss of this protein causes mitochondrial dysfunction and an increase in DNA damage after UV-C irradiation [154].

It is now becoming clear that, like nuclear DNA maintenance, mitochondria also utilise a broad range of repair and tolerance mechanisms to overcome DNA damage, which may occur spontaneously or arise due to many different environmental issues. The presence of several TLS polymerases within the organelle suggests that mitochondria also utilise such specialised polymerases to overcome DNA lesions. As well as direct extension of the replication fork, such polymerases are also likely to be vital for post-replicative repair across lesions. In many cases where the fork is stalled, replication may be reprimed beyond a lesion by PrimPol or POLRMT, allowing replication to proceed. However, a polymerase capable of bypassing the lesion is then required to fill in the gap left behind, thus allowing repair of the lesion that must be done on dsDNA to prevent the formation of double-strand breaks, which are highly deleterious for the cell.

### DNA structural barriers in mitochondria — bypass of G4 quadruplex structures

G4 quadruplexes (G4s) are stable DNA secondary structures formed through the planar stacking of quartets of Hoogsteen-bonded guanine bases. They are thought to have a wide range of roles within cells including replication initiation, telomere maintenance, epigenetic instability, regulated recombination in the immune system and transcription regulation [155]. G4s form in both DNA and RNA and can be either intramolecular, within one DNA strand, or intermolecular, between two or more DNA/RNA strands [156]. Along with the well-studied RNA/DNA quadruplex found in the NCR, *in silico* studies have identified many other potential G4-forming sequences within mtDNA [157,158]. A total of 80–90 potential quadruplex-forming sequences have been found throughout the human mitochondrial genome, strongly biased towards the heavy strand of DNA, which has a significantly high content of guanine nucleotides. Yeast mtDNA contains ∼0.373 potential quadruplex-forming sequences per 1000 base pairs, which is an order of magnitude larger than in nuclear DNA (∼0.034–0.067) [159,160]. Interestingly, some studies suggest that, along with other helix-distorting and intrinsically curved regions of DNA, G4s may be a significant cause of DNA instability within the mitochondrial genome [161]. Dong et al. [158] have identified many quadruplexes with two or three consecutive guanines within the mitochondrial genome that are associated with sites of common mitochondrial deletions. They proposed that such structures may induce genome deletions in many possible ways, including the stalling of replication, causing DNA breaks or extensive tracts of vulnerable ssDNA or through aberrant DNA repair mechanisms [158]. In addition, potential quadruplex-forming sequences are found close to sites of common pathological deletions and such sequences are inefficiently unwound by the mitochondrial helicase, Twinkle, suggesting that they may cause a significant impediment to progressing replication forks [162]. In addition, many other DNA structures may also persist within mtDNA, which may slow or stall the replicative polymerase, such as triplex or Z DNA, intrinsically bent structures or hairpin loops, such as the loop shown to form at O_L_ [161,163].

It has recently been demonstrated that PrimPol is crucial for the bypass of G4s in the nucleus of vertebrate cells [164]. Although PrimPol is unable to replicate directly through these structures, it is capable of repriming directly after G4s in a process termed ‘close-coupled repriming’ that enables replication to be restarted almost immediately downstream from G4s (Figure 2). In the absence of PrimPol, a significant amount of uncoupling of the replication fork was observed in cells as large tracts of late-replicated DNA in the region of the quadruplex on the leading strand, which was associated with the loss of epigenetic histone marks [164]. This recent discovery has led to the speculation that PrimPol is likely to play a similar role in the replication of such structures within the mitochondrial genome. Although Twinkle shows poor activity when confronted with G4 structures [162], many helicases have been found to be involved in the bypass of such structures in the nucleus, one of which (Pif1) is also localised to the mitochondria [21,165].

### Tolerance of chain-terminating nucleoside analogues

Another situation where PrimPol has recently been shown to play a vital role is in the maintenance of replication in the presence of chain-terminating nucleoside analogues (CTNAs), such as acyclovir and abacavir. Cells lacking PrimPol showed increased sensitivity to such drugs, while PrimPol was found to be able to incorporate many of these nucleotide analogues and also perform close-coupled repriming downstream from such lesions *in vitro* [40,166]. CTNAs typically lack the essential 3′ hydroxyl moiety required for phosphodiester bond formation and therefore prevent ongoing replication when incorporated into DNA by terminating strand extension. They are commonly used to control viral infections (e.g HIV), as they are readily incorporated into DNA by reverse transcriptase, significantly slowing viral replication processes. However, they do have a range of toxic side effects caused by mitochondrial toxicity (reviewed in refs [167–169]), with long-term use of azidothymidine (AZT) causing a decrease in mitochondrial DNA in skeletal muscle and cumulative mitochondrial myopathy [170,171]. More precisely, these effects have been attributed to the incorporation of these nucleoside analogues by Pol γ (reviewed in ref. [172]). Pol γ inserts nucleoside analogues with varying ease, some incorporated at concentrations similar to standard nucleotides, while others require ∼2–5-fold higher concentrations to be efficiently incorporated [173–175]. All analogues share the ability to inhibit Pol γ-mediated replication *in vitro* by preventing any further synthesis and therefore they must be rapidly removed; otherwise, ongoing replication is inhibited [174]. The attempted removal of CTNAs from the elongating DNA strand is likely to be first tackled by the exonuclease domain of Pol γ, whose role it is to check newly replicated DNA for accuracy and quickly remove any incorrectly incorporated bases to prevent mutations from being generated [88]. The exonuclease domain of Pol γ increases its fidelity by improving nucleotide selection by a factor of ∼200 [88]. Notably, removal of nucleoside analogues by this exonuclease is a slow process. For example, the ddC analogue zalcitidine has a half-life in DNA of ∼2.4 h due to its stronger binding affinity, compared with standard nucleotides, for the polymerisation domain [176]. In addition, other analogues (e.g. AZT) have also been shown to inhibit the exonucleolytic activity of Pol γ, causing a decrease in fidelity [174].

Given this propensity for incorporation of CTNAs into replicating mtDNA, it seems likely that PrimPol also plays a key role in repriming replication after the incorporation of these nucleoside analogues by Pol γ (Figure 2). PrimPol-deficient cells are more sensitive to the presence of these analogues, and this sensitivity can be complemented by the addition of PrimPol, but not by a primase-deficient mutant of this enzyme [40], indicating that repriming is essential for this process. In addition, PrimPol is capable of repriming downstream from incorporated nucleoside analogues *in vitro*, further supporting this proposed role in replication restart. Interestingly, PrimPol is itself capable of incorporating a number of the FDA-approved CTNAs into DNA, albeit less efficiently than natural nucleotides, with a distinct discrimination profile compared with Pol γ [166]. Thus, PrimPol may also create its own problems, but its low processivity [34] is likely to limit such potential toxicity *in vivo*.

## Conclusions

Despite its small size, mitochondrial DNA can represent ∼1% of the total DNA in some cells due to its polyploid nature [17]. However, our understanding of DNA replication processes within this organelle still trails well behind that of nuclear genome duplication. Mitochondrial DNA has its own specialised replicative polymerase and mechanisms that vary from those observed in the nucleus, although some controversy remains within the field over the abundance and validity of the different proposed replication models. However, we are beginning to uncover that mtDNA replication and repair mechanisms may, in fact, be more similar to those in the nucleus than was initially thought, as many proteins identified within the nucleus are now also being found to have additional roles in mtDNA replication. Clearly, there is much more to understand about the replication of this small, but far from insignificant, molecule of DNA.

PrimPol is likely to play a similar role within the mitochondrion as it does in the nucleus, maintaining the progression of the replication fork after replisome stalling. Emerging data suggest that PrimPol's key role is likely to be in repriming replication restart after a fork-stalling lesion or DNA structure [40,164]. In this review, we propose that it is highly likely that PrimPol plays the same roles in mitochondria by repriming DNA replication to allow replication to be completed in an efficient and timely manner (Figure 2).

However, mitochondrial DNA organisation varies significantly from the compaction of nuclear DNA; therefore, the replication mechanisms and proteins required for its duplication are adapted for these different environments, suggesting that PrimPol must be a highly flexible protein, which is capable of adapting its functions depending on its partners and the problems it encounters. It seems likely that its main role is to reprime the initiation of DNA synthesis after Pol γ is stalled by DNA damage, secondary structures or chain-terminating events. This allows replication to proceed, leaving behind the cause of the stalling event to be processed and corrected in a post-replicative fashion. However, its ability to perform TLS opposite template lesions, such as 8-oxoG, may also be significant [28,29]. Complete replication of a mitochondrial DNA molecule is thought to take ∼1 h [110]; thus, although each cell contains multiple copies of the mitochondrial genome, it is essential that each round of replication is completed in a timely manner to prevent the accumulation of multiple stalled and/or collapsed replication forks. Indeed, in the absence of PrimPol, the mtDNA copy number is considerably increased, suggesting that more copies are required to fully maintain organelle functionality.

Although we have primarily discussed the possible roles of PrimPol during mitochondrial genome replication, it is possible that it may also play additional roles in the repair of damaged DNA in the absence of ongoing replication. For example, almost all DNA repair pathways utilise polymerases to fill in gaps generated by nucleolytic repair processes, such as base/nucleotide excision and resection. Notably, in this regard, distinct primase–polymerases have evolved to play such bespoke roles in various DNA repair processes in prokaryotes, such as NHEJ [31,32]. We still have much more to learn about the roles and regulation of PrimPol in both the mitochondria and the nucleus, and further research is required to better understand the functions this fascinating enzyme fulfils within the cell [177]. A range of PrimPol mutations have been found in cancer cells and other conditions suggesting possible connections to human disease, including mitopathies, although these pathological associations remain to be established [178].

## Abbreviations

2D AGE: 2D neutral agarose gel electrophoresis
6-4 PP: 6-4 photoproduct
8-oxoG: 8-oxoguanine
AEP: archaeo-eukaryotic primase
AZT: azidothymidine
CSBs: conserved sequence blocks
CTNAs: chain-terminating nucleoside analogues
ds: double-stranded
G4s: G4 quadruplexes
HMG: high-mobility group
LSP and HSP: light- and heavy-strand promoters
mtDNA: mitochondria DNA
NCR: non-coding region
O_L_: the origin of light-strand DNA replication
Pol γ: polymerase γ
POLRMT: mitochondrial RNA polymerase
PrimPol: Primase–Polymerase
RITOLS: Ribonucleotide Incorporation ThroughOut the Lagging Strand
ss: single-stranded
TFAM: mitochondrial transcription factor A
TLS: translesion synthesis

## References

[1] Legros F., Malka F., Frachon P., Lombès A. and Rojo M. (2004) Organization and dynamics of human mitochondrial DNA. J. Cell Sci. 117, 2653–2662 doi:10.1242/jcs.01134 15138283

[2] Maleszka R., Skelly P.J. and Clark-Walker G.D. (1991) Rolling circle replication of DNA in yeast mitochondria. EMBO J. 10, 3923–3929 1935911 10.1002/j.1460-2075.1991.tb04962.x PMC453131

[3] Gualberto J.M., Mileshina D., Wallet C., Niazi A.K., Weber-Lotfi F. and Dietrich A. (2014) The plant mitochondrial genome: dynamics and maintenance. Biochimie 100, 107–120 doi:10.1016/j.biochi.2013.09.016 24075874

[4] Cupp J.D. and Nielsen B.L. (2014) Minireview: DNA replication in plant mitochondria. Mitochondrion 19(Pt B), 231–237 doi:10.1016/j.mito.2014.03.008 24681310 PMC4177014

[5] Lukeš J., Lys Guilbride D., Votýpka J., Zíková A., Benne R. and Englund P.T. (2002) Kinetoplast DNA network: evolution of an improbable structure. Eukaryot. Cell 1, 495–502 doi:10.1128/EC.1.4.495-502.2002 12455998 PMC117999

[6] Jensen R.E. and Englund P.T. (2012) Network news: the replication of kinetoplast DNA. Annu. Rev. Microbiol. 66, 473–491 doi:10.1146/annurev-micro-092611-150057 22994497

[7] Iborra F.J., Kimura H. and Cook P.R. (2004) The functional organization of mitochondrial genomes in human cells. BMC Biol. 2, 9 doi:10.1186/1741-7007-2-9 15157274 PMC425603

[8] Kukat C., Wurm C.A., Spahr H., Falkenberg M., Larsson N.-G. and Jakobs S. (2011) Super-resolution microscopy reveals that mammalian mitochondrial nucleoids have a uniform size and frequently contain a single copy of mtDNA. Proc. Natl Acad. Sci. U.S.A. 108, 13534–13539 doi:10.1073/pnas.1109263108 21808029 PMC3158146

[9] Gerhold J.M., Cansiz-Arda Ş., Lõhmus M., Engberg O., Reyes A., van Rennes H. et al. (2015) Human mitochondrial DNA-protein complexes attach to a cholesterol-rich membrane structure. Sci. Rep. 5, 15292 doi:10.1038/srep15292 26478270 PMC4609938

[10] Kasashima K. and Endo H. (2015) Interaction of human mitochondrial transcription factor A in mitochondria: its involvement in the dynamics of mitochondrial DNA nucleoids. Genes Cells 20, 1017–1027 doi:10.1111/gtc.12306 26445116

[11] Campbell C.T., Kolesar J.E. and Kaufman B.A. (2012) Mitochondrial transcription factor A regulates mitochondrial transcription initiation, DNA packaging, and genome copy number. Biochim. Biophys. Acta, Gene Regul. Mech. 1819, 921–929 doi:10.1016/j.bbagrm.2012.03.002 22465614

[12] Kasamatsu H., Robberson D.L. and Vinograd J. (1971) A novel closed-circular mitochondrial DNA with properties of a replicating intermediate. Proc. Natl Acad. Sci. U.S.A. 68, 2252–2257 doi:10.1073/pnas.68.9.2252 5289384 PMC389395

[13] Walberg M.W. and Clayton D.A. (1981) Sequence and properties of the human KB cell and mouse L cell D-loop regions of mitochondrial DNA. Nucleic Acids Res. 9, 5411–5421 doi:10.1093/nar/9.20.5411 7301592 PMC327529

[14] Nicholls T.J. and Minczuk M. (2014) In D-loop: 40 years of mitochondrial 7S DNA. Exp. Gerontol. 56, 175–181 doi:10.1016/j.exger.2014.03.027 24709344

[15] Brown W.M., Shine J. and Goodman H.M. (1978) Human mitochondrial DNA: analysis of 7S DNA from the origin of replication. Proc. Natl Acad. Sci. U.S.A. 75, 735–739 doi:10.1073/pnas.75.2.735 273237 PMC411331

[16] He J., Mao C.-C., Reyes A., Sembongi H., Di Re M., Granycome C. et al. (2007) The AAA^+^ protein ATAD3 has displacement loop binding properties and is involved in mitochondrial nucleoid organization. J. Cell Biol. 176, 141–146 doi:10.1083/jcb.200609158 17210950 PMC2063933

[17] Holt I.J., He J., Mao C.-C., Boyd-Kirkup J.D., Martinsson P., Sembongi H. et al. (2007) Mammalian mitochondrial nucleoids: organizing an independently minded genome. Mitochondrion 7, 311–321 doi:10.1016/j.mito.2007.06.004 17698423

[18] Antes A., Tappin I., Chung S., Lim R., Lu B., Parrott A.M. et al. (2010) Differential regulation of full-length genome and a single-stranded 7S DNA along the cell cycle in human mitochondria. Nucleic Acids Res. 38, 6466–6476 doi:10.1093/nar/gkq493 20530535 PMC2965228

[19] Akman G., Desai R., Bailey L.J., Yasukawa T., Dalla Rosa I., Durigon R. et al. (2016) Pathological ribonuclease H1 causes R-loop depletion and aberrant DNA segregation in mitochondria. Proc. Natl Acad. Sci. U.S.A. 113, E4276–E4285 doi:10.1073/pnas.1600537113 27402764 PMC4968715

[20] Liu P., Qian L., Sung J.-S., de Souza-Pinto N.C., Zheng L., Bogenhagen D.F. et al. (2008) Removal of oxidative DNA damage via FEN1-dependent long-patch base excision repair in human cell mitochondria. Mol. Cell. Biol. 28, 4975–4987 doi:10.1128/MCB.00457-08 18541666 PMC2519700

[21] Kazak L., Reyes A., Duncan A.L., Rorbach J., Wood S.R., Brea-Calvo G. et al. (2013) Alternative translation initiation augments the human mitochondrial proteome. Nucleic Acids Res. 41, 2354–2369 doi:10.1093/nar/gks1347 23275553 PMC3575844

[22] Lahaye A., Stahl H., Thines-Sempoux D. and Foury F. (1991) PIF1: a DNA helicase in yeast mitochondria. EMBO J. 10, 997–1007 1849081 10.1002/j.1460-2075.1991.tb08034.x PMC452744

[23] Duxin J.P., Dao B., Martinsson P., Rajala N., Guittat L., Campbell J.L. et al. (2009) Human Dna2 is a nuclear and mitochondrial DNA maintenance protein. Mol. Cell. Biol. 29, 4274–4282 doi:10.1128/MCB.01834-08 19487465 PMC2715806

[24] Zheng L., Zhou M., Guo Z., Lu H., Qian L., Dai H. et al. (2008) Human DNA2 is a mitochondrial nuclease/helicase for efficient processing of DNA replication and repair intermediates. Mol. Cell 32, 325–336 doi:10.1016/j.molcel.2008.09.024 18995831 PMC2636562

[25] Kazak L., Reyes A., He J., Wood S.R., Brea-Calvo G., Holen T.T. et al. (2013) A cryptic targeting signal creates a mitochondrial FEN1 isoform with tailed R-Loop binding properties. PLoS ONE 8, e62340 doi:10.1371/journal.pone.0062340 23675412 PMC3652857

[26] Sage J.M., Gildemeister O.S. and Knight K.L. (2010) Discovery of a novel function for human Rad51: maintenance of the mitochondrial genome. J. Biol. Chem. 285, 18984–18990 doi:10.1074/jbc.M109.099846 20413593 PMC2885175

[27] Iyer L.M., Koonin E.V., Leipe D.D. and Aravind L. (2005) Origin and evolution of the archaeo-eukaryotic primase superfamily and related palm-domain proteins: structural insights and new members. Nucleic Acids Res. 33, 3875–3896 doi:10.1093/nar/gki702 16027112 PMC1176014

[28] Bianchi J., Rudd S.G., Jozwiakowski S.K., Bailey L.J., Soura V., Taylor E. et al. (2013) PrimPol bypasses UV photoproducts during eukaryotic chromosomal DNA replication. Mol. Cell 52, 566–573 doi:10.1016/j.molcel.2013.10.035 24267451 PMC4228047

[29] García-Gómez S., Reyes A., Martínez-Jiménez M.I., Chocrón E.S., Mourón S., Terrados G. et al. (2013) PrimPol, an archaic primase/polymerase operating in human cells. Mol. Cell 52, 541–553 doi:10.1016/j.molcel.2013.09.025 24207056 PMC3899013

[30] Wan L., Lou J., Xia Y., Su B., Liu T., Cui J. et al. (2013) hPrimpol1/CCDC111 is a human DNA primase-polymerase required for the maintenance of genome integrity. EMBO Rep. 14, 1104–1112 doi:10.1038/embor.2013.159 24126761 PMC3981091

[31] Rudd S.G., Bianchi J. and Doherty A.J. (2014) PrimPol — a new polymerase on the block. Mol. Cell. Oncol. 1, e960754 doi:10.4161/23723548.2014.960754 27308331 PMC4905188

[32] Guilliam T.A., Keen B.A., Brissett N.C. and Doherty A.J. (2015) Primase-polymerases are a functionally diverse superfamily of replication and repair enzymes. Nucleic Acids Res. 43, 6651–6664 doi:10.1093/nar/gkv625 26109351 PMC4538821

[33] Tokarsky E.J., Wallenmeyer P.C., Phi K.K. and Suo Z. (2017) Significant impact of divalent metal ions on the fidelity, sugar selectivity, and drug incorporation efficiency of human PrimPol. DNA Repair 49, 51–59 doi:10.1016/j.dnarep.2016.11.003 27989484 PMC8225440

[34] Keen B.A., Jozwiakowski S.K., Bailey L.J., Bianchi J. and Doherty A.J. (2014) Molecular dissection of the domain architecture and catalytic activities of human PrimPol. Nucleic Acids Res. 42, 5830–5845 doi:10.1093/nar/gku214 24682820 PMC4027207

[35] Guilliam T.A., Jozwiakowski S.K., Ehlinger A., Barnes R.P., Rudd S.G., Bailey L.J. et al. (2015) Human PrimPol is a highly error-prone polymerase regulated by single-stranded DNA binding proteins. Nucleic Acids Res. 43, 1056–1068 doi:10.1093/nar/gku1321 25550423 PMC4333378

[36] Martínez-Jiménez M.I., García-Gómez S., Bebenek K., Sastre-Moreno G., Calvo P.A., Díaz-Talavera A. et al. (2015) Alternative solutions and new scenarios for translesion DNA synthesis by human PrimPol. DNA Repair 29, 127–138 doi:10.1016/j.dnarep.2015.02.013 25746449

[37] Rechkoblit O., Gupta Y.K., Malik R., Rajashankar K.R., Johnson R.E., Prakash L. et al. (2016) Structure and mechanism of human PrimPol, a DNA polymerase with primase activity. Sci. Adv. 2, e1601317 doi:10.1126/sciadv.1601317 27819052 PMC5088642

[38] Rudd S.G., Glover L., Jozwiakowski S.K., Horn D. and Doherty A.J. (2013) PPL2 translesion polymerase is essential for the completion of chromosomal DNA replication in the African trypanosome. Mol. Cell 52, 554–565 doi:10.1016/j.molcel.2013.10.034 24267450 PMC3898837

[39] Bailey L.J., Bianchi J., Hégarat N., Hochegger H. and Doherty A.J. (2016) PrimPol-deficient cells exhibit a pronounced G2 checkpoint response following UV damage. Cell Cycle 15, 908–918 doi:10.1080/15384101.2015.1128597 26694751 PMC4889237

[40] Kobayashi K., Guilliam T.A., Tsuda M., Yamamoto J., Bailey L.J., Iwai S. et al. (2016) Repriming by PrimPol is critical for DNA replication restart downstream of lesions and chain-terminating nucleosides. Cell Cycle 15, 1997–2008 doi:10.1080/15384101.2016.1191711 27230014 PMC4968974

[41] Bianchi J. (2013) Investigating the Role of a Novel Primase-Polymerase, PrimPol, in DNA Damage Tolerance in Vertebrate Cells. Ph.D. Thesis, University of Sussex

[42] Guilliam T.A., Bailey L.J., Brissett N.C. and Doherty A.J. (2016) PolDIP2 interacts with human PrimPol and enhances its DNA polymerase activities. Nucleic Acids Res. 44, 3317–3329 doi:10.1093/nar/gkw175 26984527 PMC4838387

[43] Stojkovič G., Makarova A.V., Wanrooij P.H., Forslund J., Burgers P.M. and Wanrooij S. (2016) Oxidative DNA damage stalls the human mitochondrial replisome. Sci. Rep. 6, 28942 doi:10.1038/srep28942 27364318 PMC4929447

[44] Pellegrini L. (2012) The Pol α-primase complex. Subcell. Biochem. 62, 157–169 doi:10.1007/978-94-007-4572-8_9 22918585

[45] Pellegrini L. and Costa A. (2016) New insights into the mechanism of DNA duplication by the eukaryotic replisome. Trends Biochem. Sci. 41, 859–871 doi:10.1016/j.tibs.2016.07.011 27555051

[46] Wong T.W. and Clayton D.A. (1985) Isolation and characterization of a DNA primase from human mitochondria. J. Biol. Chem. 260, 11530–11535 4044569

[47] Ledwith B.J., Manam S. and Van Tuyle G.C. (1986) Characterization of a DNA primase from rat liver mitochondria. J. Biol. Chem. 261, 6571–6577 3700409

[48] Wong T.W. and Clayton D.A. (1986) DNA primase of human mitochondria is associated with structural RNA that is essential for enzymatic activity. Cell 45, 817–825 doi:10.1016/0092-8674(86)90556-8 3011281

[49] Wanrooij S., Fuste J.M., Farge G., Shi Y., Gustafsson C.M. and Falkenberg M. (2008) Human mitochondrial RNA polymerase primes lagging-strand DNA synthesis in vitro. Proc. Natl Acad. Sci. U.S.A. 105, 11122–11127 doi:10.1073/pnas.0805399105 18685103 PMC2516254

[50] Masters B.S., Stohl L.L. and Clayton D.A. (1987) Yeast mitochondrial RNA polymerase is homologous to those encoded by bacteriophages T3 and T7. Cell 51, 89–99 doi:10.1016/0092-8674(87)90013-4 3308116

[51] Tiranti V., Savoia A., Forti F., D'Apolito M.F., Centra M., Rocchi M. et al. (1997) Identification of the gene encoding the human mitochondrial RNA polymerase (h-mtRPOL) by cyberscreening of the Expressed Sequence Tags database. Hum. Mol. Genet. 6, 615–625 doi:10.1093/hmg/6.4.615 9097968

[52] Arnold J.J., Sharma S.D., Feng J.Y., Ray A.S., Smidansky E.D., Kireeva M.L. et al. (2012) Sensitivity of mitochondrial transcription and resistance of RNA polymerase II dependent nuclear transcription to antiviral ribonucleosides. PLoS Pathog. 8, e1003030 doi:10.1371/journal.ppat.1003030 23166498 PMC3499576

[53] Fischer H. and Hinkle D.C. (1980) Bacteriophage T7 DNA replication in vitro. Stimulation of DNA synthesis by T7 RNA polymerase. J. Biol. Chem. 255, 7956–7964 6249822

[54] Hinkle D.C. (1980) Evidence for direct involvement of T7 RNA polymerase bacteriophage DNA replication. J. Virol. 34, 136–141 7373707 10.1128/jvi.34.1.136-141.1980 PMC288679

[55] Romano L.J., Tamanoi F. and Richardson C.C. (1981) Initiation of DNA replication at the primary origin of bacteriophage T7 by purified proteins: requirement for T7 RNA polymerase. Proc. Natl Acad. Sci. U.S.A. 78, 4107–4111 doi:10.1073/pnas.78.7.4107 6945573 PMC319735

[56] Posse V., Hoberg E., Dierckx A., Shahzad S., Koolmeister C., Larsson N.-G. et al. (2014) The amino terminal extension of mammalian mitochondrial RNA polymerase ensures promoter specific transcription initiation. Nucleic Acids Res. 42, 3638–3647 doi:10.1093/nar/gkt1397 24445803 PMC3973307

[57] Litonin D., Sologub M., Shi Y., Savkina M., Anikin M., Falkenberg M. et al. (2010) Human mitochondrial transcription revisited: only TFAM and TFB2M are required for transcription of the mitochondrial genes in vitro. J. Biol. Chem. 285, 18129–18133 doi:10.1074/jbc.C110.128918 20410300 PMC2881736

[58] Ramachandran A., Basu U., Sultana S., Nandakumar D. and Patel S.S. (2017) Human mitochondrial transcription factors TFAM and TFB2M work synergistically in promoter melting during transcription initiation. Nucleic Acids Res. 45, 861–874 doi:10.1093/nar/gkw1157 27903899 PMC5314767

[59] Posse V. and Gustafsson C.M. (2017) Human mitochondrial transcription factor B2 is required for promoter melting during initiation of transcription. J. Biol. Chem. 292, 2637–2645 doi:10.1074/jbc.M116.751008 28028173 PMC5314162

[60] Morozov Y.I., Parshin A.V., Agaronyan K., Cheung A.C.M., Anikin M., Cramer P. et al. (2015) A model for transcription initiation in human mitochondria. Nucleic Acids Res. 43, 3726–3735 doi:10.1093/nar/gkv235 25800739 PMC4402542

[61] Falkenberg M., Gaspari M., Rantanen A., Trifunovic A., Larsson N.-G. and Gustafsson C.M. (2002) Mitochondrial transcription factors B1 and B2 activate transcription of human mtDNA. Nat. Genet. 31, 289–294 doi:10.1038/ng909 12068295

[62] Deshpande A.P. and Patel S.S. (2012) Mechanism of transcription initiation by the yeast mitochondrial RNA polymerase. Biochim. Biophys. Acta, Gene Regul. Mech. 1819, 930–938 doi:10.1016/j.bbagrm.2012.02.003 PMC3381941 22353467

[63] Kuhl I., Miranda M., Posse V., Milenkovic D., Mourier A., Siira S.J. et al. (2016) POLRMT regulates the switch between replication primer formation and gene expression of mammalian mtDNA. Sci. Adv. 2, e1600963 doi:10.1126/sciadv.1600963 27532055 PMC4975551

[64] Gillum A.M. and Clayton D.A. (1979) Mechanism of mitochondrial DNA replication in mouse L-cells: RNA priming during the initiation of heavy-strand synthesis. J. Mol. Biol. 135, 353–368 doi:10.1016/0022-2836(79)90441-8 537082

[65] Korhonen J.A., Pham X.H., Pellegrini M. and Falkenberg M. (2004) Reconstitution of a minimal mtDNA replisome in vitro. EMBO J. 23, 2423–2429 doi:10.1038/sj.emboj.7600257 15167897 PMC423294

[66] Pham X.H., Farge G., Shi Y., Gaspari M., Gustafsson C.M. and Falkenberg M. (2006) Conserved sequence box II directs transcription termination and primer formation in mitochondria. J. Biol. Chem. 281, 24647–24652 doi:10.1074/jbc.M602429200 16790426

[67] Montoya J., Christianson T., Levens D., Rabinowitz M. and Attardi G. (1982) Identification of initiation sites for heavy-strand and light-strand transcription in human mitochondrial DNA. Proc. Natl Acad. Sci. U.S.A. 79, 7195–7199 doi:10.1073/pnas.79.23.7195 6185947 PMC347305

[68] Cantatore P. and Attardi G. (1980) Mapping of nascent light and heavy strand transcripts on the physical map of HeLa cell mitochondrial DNA. Nucleic Acids Res. 8, 2605–2626 doi:10.1093/nar/8.12.2605 6159578 PMC324109

[69] Xu B. and Clayton D.A. (1996) RNA-DNA hybrid formation at the human mitochondrial heavy-strand origin ceases at replication start sites: an implication for RNA-DNA hybrids serving as primers. EMBO J. 15, 3135–3143 8670814 PMC450256

[70] Xu B. and Clayton D.A. (1995) A persistent RNA-DNA hybrid is formed during transcription at a phylogenetically conserved mitochondrial DNA sequence. Mol. Cell. Biol. 15, 580–589 doi:10.1128/MCB.15.1.580 7528331 PMC232017

[71] Lee D.Y. and Clayton D.A. (1996) Properties of a primer RNA-DNA hybrid at the mouse mitochondrial DNA leading-strand origin of replication. J. Biol. Chem. 271, 24262–24269 doi:10.1074/jbc.271.39.24262 8798672

[72] Lee D.Y. and Clayton D.A. (1997) RNase mitochondrial RNA processing correctly cleaves a novel R loop at the mitochondrial DNA leading-strand origin of replication. Genes Dev. 11, 582–592 doi:10.1101/gad.11.5.582 9119223

[73] Wanrooij P.H., Uhler J.P., Shi Y., Westerlund F., Falkenberg M. and Gustafsson C.M. (2012) A hybrid G-quadruplex structure formed between RNA and DNA explains the extraordinary stability of the mitochondrial R-loop. Nucleic Acids Res. 40, 10334–10344 doi:10.1093/nar/gks802 22965135 PMC3488243

[74] Zheng K.-W., Wu R.-Y., He Y.-D., Xiao S., Zhang J.-Y., Liu J.-Q. et al. (2014) A competitive formation of DNA:RNA hybrid G-quadruplex is responsible to the mitochondrial transcription termination at the DNA replication priming site. Nucleic Acids Res. 42, 10832–10844 doi:10.1093/nar/gku764 25140009 PMC4176368

[75] Tan B.G., Wellesley F.C., Savery N.J. and Szczelkun M.D. (2016) Length heterogeneity at conserved sequence block 2 in human mitochondrial DNA acts as a rheostat for RNA polymerase POLRMT activity. Nucleic Acids Res. 44, 7817–7829 doi:10.1093/nar/gkw648 27436287 PMC5027508

[76] Minczuk M., He J., Duch A.M., Ettema T.J., Chlebowski A., Dzionek K. et al. (2011) TEFM (c17orf42) is necessary for transcription of human mtDNA. Nucleic Acids Res. 39, 4284–4299 doi:10.1093/nar/gkq1224 21278163 PMC3105396

[77] Agaronyan K., Morozov Y.I., Anikin M. and Temiakov D. (2015) Replication-transcription switch in human mitochondria. Science 347, 548–551 doi:10.1126/science.aaa0986 25635099 PMC4677687

[78] Fish J., Raule N. and Attardi G. (2004) Discovery of a major D-loop replication origin reveals two modes of human mtDNA synthesis. Science 306, 2098–2101 doi:10.1126/science.1102077 15604407

[79] Yasukawa T., Reyes A., Cluett T.J., Yang M.-Y., Bowmaker M., Jacobs H.T. et al. (2006) Replication of vertebrate mitochondrial DNA entails transient ribonucleotide incorporation throughout the lagging strand. EMBO J. 25, 5358–5371 doi:10.1038/sj.emboj.7601392 17066082 PMC1636616

[80] Anderson S., Bankier A.T., Barrell B.G., de Bruijn M.H.L., Coulson A.R., Drouin J. et al. (1981) Sequence and organization of the human mitochondrial genome. Nature 290, 457–465 doi:10.1038/290457a0 7219534

[81] Fusté J.M., Wanrooij S., Jemt E., Granycome C.E., Cluett T.J., Shi Y. et al. (2010) Mitochondrial RNA polymerase is needed for activation of the origin of light-strand DNA replication. Mol. Cell 37, 67–78 doi:10.1016/j.molcel.2009.12.021 20129056

[82] Wanrooij S., Miralles Fusté J., Stewart J.B., Wanrooij P.H., Samuelsson T., Larsson N.-G. et al. (2012) In vivo mutagenesis reveals that OriL is essential for mitochondrial DNA replication. EMBO Rep. 13, 1130–1137 doi:10.1038/embor.2012.161 23090476 PMC3513414

[83] Kaguni L.S. (2004) DNA polymerase γ, the mitochondrial replicase. Annu. Rev. Biochem. 73, 293–320 doi:10.1146/annurev.biochem.72.121801.161455 15189144

[84] Bolden A., Noy G.P. and Weissbach A. (1977) DNA polymerase of mitochondria is a gamma-polymerase. J. Biol. Chem. 252, 3351–3356 16896

[85] Wernette C.M. and Kaguni L.S. (1986) A mitochondrial DNA polymerase from embryos of *Drosophila melanogaster*. Purification, subunit structure, and partial characterization. J. Biol. Chem. 261, 14764–14770 3095323

[86] Shutt T.E. and Gray M.W. (2006) Bacteriophage origins of mitochondrial replication and transcription proteins. Trends Genet. 22, 90–95 doi:10.1016/j.tig.2005.11.007 16364493

[87] Hance N., Ekstrand M.I. and Trifunovic A. (2005) Mitochondrial DNA polymerase gamma is essential for mammalian embryogenesis. Hum. Mol. Genet. 14, 1775–1783 doi:10.1093/hmg/ddi184 15888483

[88] Johnson A.A. and Johnson K.A. (2001) Exonuclease proofreading by human mitochondrial DNA polymerase. J. Biol. Chem. 276, 38097–38107 11477094 10.1074/jbc.M106046200

[89] Johnson A.A. and Johnson K.A. (2001) Fidelity of nucleotide incorporation by human mitochondrial DNA polymerase. J. Biol. Chem. 276, 38090–38096 11477093 10.1074/jbc.M106045200

[90] Graziewicz M.A., Longley M.J. and Copeland W.C. (2006) DNA polymerase γ in mitochondrial DNA replication and repair. Chem. Rev. 106, 383–405 doi:10.1021/cr040463d 16464011

[91] Lee Y.-S., Kennedy W.D. and Yin Y.W. (2009) Structural insight into processive human mitochondrial DNA synthesis and disease-related polymerase mutations. Cell 139, 312–324 doi:10.1016/j.cell.2009.07.050 19837034 PMC3018533

[92] Szymanski M.R., Kuznetsov V.B., Shumate C., Meng Q., Lee Y.-S., Patel G. et al. (2015) Structural basis for processivity and antiviral drug toxicity in human mitochondrial DNA replicase. EMBO J. 34, 1959–1970 doi:10.15252/embj.201591520 26056153 PMC4547898

[93] Stumpf J.D. and Copeland W.C. (2011) Mitochondrial DNA replication and disease: insights from DNA polymerase γ mutations. Cell. Mol. Life Sci. 68, 219–233 doi:10.1007/s00018-010-0530-4 20927567 PMC3046768

[94] Carrodeguas J.A., Theis K., Bogenhagen D.F. and Kisker C. (2001) Crystal structure and deletion analysis show that the accessory subunit of mammalian DNA polymerase γ, PolγB, functions as a homodimer. Mol. Cell 7, 43–54 doi:10.1016/S1097-2765(01)00153-8 11172710

[95] Carrodeguas J.A., Pinz K.G. and Bogenhagen D.F. (2002) DNA binding properties of human pol γB. J. Biol. Chem. 277, 50008–50014 doi:10.1074/jbc.M207030200 12379656

[96] Farge G., Pham X.H., Holmlund T., Khorostov I. and Falkenberg M. (2007) The accessory subunit B of DNA polymerase γ is required for mitochondrial replisome function. Nucleic Acids Res. 35, 902–911 doi:10.1093/nar/gkl1116 PMC1807957 17251196

[97] Fan L., Sanschagrin P.C., Kaguni L.S. and Kuhn L.A. (1999) The accessory subunit of mtDNA polymerase shares structural homology with aminoacyl-tRNA synthetases: implications for a dual role as a primer recognition factor and processivity clamp. Proc. Natl Acad. Sci. U.S.A. 96, 9527–9532 doi:10.1073/pnas.96.17.9527 10449726 PMC22242

[98] Croteau D.L., Rossi M.L., Canugovi C., Tian J., Sykora P., Ramamoorthy M. et al. (2012) RECQL4 localizes to mitochondria and preserves mitochondrial DNA integrity. Aging Cell 11, 456–466 doi:10.1111/j.1474-9726.2012.00803.x 22296597 PMC3350572

[99] Wang Y., Lyu Y.L. and Wang J.C. (2002) Dual localization of human DNA topoisomerase IIIα to mitochondria and nucleus. Proc. Natl Acad. Sci. U.S.A. 99, 12114–12119 doi:10.1073/pnas.192449499 12209014 PMC129407

[100] Low R.L., Orton S. and Friedman D.B. (2003) A truncated form of DNA topoisomerase IIβ associates with the mtDNA genome in mammalian mitochondria. Eur. J. Biochem. 270, 4173–4186 doi:10.1046/j.1432-1033.2003.03814.x 14519130

[101] Zhang H., Barcelo J.M., Lee B., Kohlhagen G., Zimonjic D.B., Popescu N.C. et al. (2001) Human mitochondrial topoisomerase I. Proc. Natl Acad. Sci. U.S.A. 98, 10608–10613 doi:10.1073/pnas.191321998 11526219 PMC58513

[102] Lakshmipathy U. and Campbell C. (1999) The human DNA ligase III gene encodes nuclear and mitochondrial proteins. Mol. Cell. Biol. 19, 3869–3876 doi:10.1128/MCB.19.5.3869 10207110 PMC84244

[103] Ruhanen H., Ushakov K. and Yasukawa T. (2011) Involvement of DNA ligase III and ribonuclease H1 in mitochondrial DNA replication in cultured human cells. Biochim. Biophys. Acta, Mol. Cell Res. 1813, 2000–2007 doi:10.1016/j.bbamcr.2011.08.008 PMC3223524 21878356

[104] Cerritelli S.M., Frolova E.G., Feng C., Grinberg A., Love P.E. and Crouch R.J. (2003) Failure to produce mitochondrial DNA results in embryonic lethality in Rnaseh1 null mice. Mol. Cell 11, 807–815 doi:10.1016/S1097-2765(03)00088-1 12667461

[105] Reyes A., Melchionda L., Nasca A., Carrara F., Lamantea E., Zanolini A. et al. (2015) RNASEH1 mutations impair mtDNA replication and cause adult-onset mitochondrial encephalomyopathy. Am. J. Hum. Genet. 97, 186–193 doi:10.1016/j.ajhg.2015.05.013 26094573 PMC4572567

[106] Holmes J.B., Akman G., Wood S.R., Sakhuja K., Cerritelli S.M., Moss C. et al. (2015) Primer retention owing to the absence of RNase H1 is catastrophic for mitochondrial DNA replication. Proc. Natl Acad. Sci. U.S.A. 112, 9334–9339 doi:10.1073/pnas.1503653112 26162680 PMC4522766

[107] Robberson D.L., Kasamatsu H. and Vinograd J. (1972) Replication of mitochondrial DNA. Circular replicative intermediates in mouse L cells. Proc. Natl Acad. Sci. U.S.A. 69, 737–741 doi:10.1073/pnas.69.3.737 4501588 PMC426547

[108] Wolstenholme D.R., Koike K. and Cochran-Fouts P. (1973) Single strand-containing replicating molecules of circular mitochondrial DNA. J. Cell Biol. 56, 230–245 doi:10.1083/jcb.56.1.230 4345165 PMC2108830

[109] Goddard J.M. and Wolstenholme D.R. (1980) Origin and direction of replication in mitochondrial DNA molecules from the genus *Drosophila*. Nucleic Acids Res. 8, 741–757 6253922 PMC327307

[110] Berk A.J. and Clayton D.A. (1974) Mechanism of mitochondrial DNA replication in mouse L-cells: asynchronous replication of strands, segregation of circular daughter molecules, aspects of topology and turnover of an initiation sequence. J. Mol. Biol. 86, 801–824 doi:10.1016/0022-2836(74)90355-6 4473554

[111] Robberson D.L. and Clayton D.A. (1972) Replication of mitochondrial DNA in mouse L cells and their thymidine kinase^−^ derivatives: displacement replication on a covalently-closed circular template. Proc. Natl Acad. Sci. U.S.A. 69, 3810–3814 doi:10.1073/pnas.69.12.3810 4509344 PMC389878

[112] Brown T.A., Cecconi C., Tkachuk A.N., Bustamante C. and Clayton D.A. (2005) Replication of mitochondrial DNA occurs by strand displacement with alternative light-strand origins, not via a strand-coupled mechanism. Genes Dev. 19, 2466–2476 doi:10.1101/gad.1352105 16230534 PMC1257401

[113] Brewer B.J., Sena E.P. and Fangman W.L. (1988) Analysis of Replication Intermediates by Two-dimensional Agarose Gel Electrophoresis In Cancer Cells, Cold Spring Harbor Laboratory (T.a.S. Kelly B., ed.), pp. 229–234, Cold Spring Harbor, NY

[114] Bowmaker M., Yang M.Y., Yasukawa T., Reyes A., Jacobs H.T., Huberman J.A. et al. (2003) Mammalian mitochondrial DNA replicates bidirectionally from an initiation zone. J. Biol. Chem. 278, 50961–50969 doi:10.1074/jbc.M308028200 14506235

[115] Holt I.J., Lorimer H.E. and Jacobs H.T. (2000) Coupled leading- and lagging-strand synthesis of mammalian mitochondrial DNA. Cell 100, 515–524 doi:10.1016/S0092-8674(00)80688-1 10721989

[116] Reyes A., Yang M.Y., Bowmaker M. and Holt I.J. (2005) Bidirectional replication initiates at sites throughout the mitochondrial genome of birds. J. Biol. Chem. 280, 3242–3250 doi:10.1074/jbc.M411916200 15557283

[117] Yang M.Y., Bowmaker M., Reyes A., Vergani L., Angeli P., Gringeri E. et al. (2002) Biased incorporation of ribonucleotides on the mitochondrial L-strand accounts for apparent strand-asymmetric DNA replication. Cell 111, 495–505 doi:10.1016/S0092-8674(02)01075-9 12437923

[118] Koike K. and Wolstenholme D.R. (1974) Evidence for discontinuous replication of circular mitochondrial DNA molecules from Novikoff rat ascites hepatoma cells. J. Cell Biol. 61, 14–25 doi:10.1083/jcb.61.1.14 4362136 PMC2109274

[119] Kirschner R.H., Wolstenholme D.R. and Gross N.J. (1968) Replicating molecules of circular mitochondrial DNA. Proc. Natl Acad. Sci. U.S.A. 60, 1466–1472 doi:10.1073/pnas.60.4.1466 5244753 PMC224942

[120] Reyes A., Kazak L., Wood S.R., Yasukawa T., Jacobs H.T. and Holt I.J. (2013) Mitochondrial DNA replication proceeds via a ‘bootlace’ mechanism involving the incorporation of processed transcripts. Nucleic Acids Res. 41, 5837–5850 doi:10.1093/nar/gkt196 23595151 PMC3675460

[121] Pohjoismäki J.L.O., Holmes J.B., Wood S.R., Yang M.-Y., Yasukawa T., Reyes A. et al. (2010) Mammalian mitochondrial DNA replication intermediates are essentially duplex but contain extensive tracts of RNA/DNA hybrid. J. Mol. Biol. 397, 1144–1155 doi:10.1016/j.jmb.2010.02.029 20184890 PMC2857715

[122] Yasukawa T., Yang M.-Y., Jacobs H.T. and Holt I.J. (2005) A bidirectional origin of replication maps to the major noncoding region of human mitochondrial DNA. Mol. Cell 18, 651–662 doi:10.1016/j.molcel.2005.05.002 15949440

[123] Miralles Fusté J., Shi Y., Wanrooij S., Zhu X., Jemt E., Persson Ö. et al. (2014) In vivo occupancy of mitochondrial single-stranded DNA binding protein supports the strand displacement mode of DNA replication. PLoS Genet. 10, e1004832 doi:10.1371/journal.pgen.1004832 25474639 PMC4256270

[124] Brown T.A., Tkachuk A.N. and Clayton D.A. (2008) Native R-loops persist throughout the mouse mitochondrial DNA genome. J. Biol. Chem. 283, 36743–36751 doi:10.1074/jbc.M806174200 18986989 PMC2605977

[125] Clayton D.A., Doda J.N. and Friedberg E.C. (1974) The absence of a pyrimidine dimer repair mechanism in mammalian mitochondria. Proc. Natl Acad. Sci. U.S.A. 71, 2777–2781 doi:10.1073/pnas.71.7.2777 4212385 PMC388554

[126] Birch-Machin M.A., Russell E.V. and Latimer J.A. (2013) Mitochondrial DNA damage as a biomarker for ultraviolet radiation exposure and oxidative stress. Br. J. Dermatol. 169(Suppl 2), 9–14 doi:10.1111/bjd.12207 23786615

[127] Rastogi R.P., Richa R.P., Kumar A., Tyagi M.B. and Sinha R.P. (2010) Molecular mechanisms of ultraviolet radiation-induced DNA damage and repair. J. Nucleic Acids 2010, 32 doi:10.4061/2010/592980 PMC3010660 21209706

[128] Kasiviswanathan R., Gustafson M.A., Copeland W.C. and Meyer J.N. (2012) Human mitochondrial DNA polymerase γ exhibits potential for bypass and mutagenesis at UV-induced cyclobutane thymine dimers. J. Biol. Chem. 287, 9222–9229 doi:10.1074/jbc.M111.306852 22194617 PMC3308766

[129] Torregrosa-Munumer R., Goffart S., Haikonen J.A. and Pohjoismaki J.L.O. (2015) Low doses of ultraviolet radiation and oxidative damage induce dramatic accumulation of mitochondrial DNA replication intermediates, fork regression, and replication initiation shift. Mol. Biol. Cell 26, 4197–4208 doi:10.1091/mbc.E15-06-0390 26399294 PMC4642854

[130] Sanchez-Sandoval E., Diaz-Quezada C., Velazquez G., Arroyo-Navarro L.F., Almanza-Martinez N., Trasvina-Arenas C.H. et al. (2015) Yeast mitochondrial RNA polymerase primes mitochondrial DNA polymerase at origins of replication and promoter sequences. Mitochondrion 24, 22–31 doi:10.1016/j.mito.2015.06.004 26184436

[131] Ramachandran A., Nandakumar D., Deshpande A.P., Lucas T.P., R-Bhojappa R., Tang G.-Q. et al. (2016) The yeast mitochondrial RNA polymerase and transcription factor complex catalyzes efficient priming of DNA synthesis on single-stranded DNA. J. Biol. Chem. 291, 16828–16839 doi:10.1074/jbc.M116.740282 27311715 PMC4974394

[132] Foury F. (1989) Cloning and sequencing of the nuclear gene MIP1 encoding the catalytic subunit of the yeast mitochondrial DNA polymerase. J. Biol. Chem. 264, 20552–20560 2684980

[133] Lecrenier N., Van Der Bruggen P. and Foury F. (1997) Mitochondrial DNA polymerases from yeast to man: a new family of polymerases. Gene 185, 147–152 doi:10.1016/S0378-1119(96)00663-4 9034326

[134] Lasserre J.-P., Plissonneau J., Velours C., Bonneu M., Litvak S., Laquel P. et al. (2013) Biochemical, cellular and molecular identification of DNA polymerase α in yeast mitochondria. Biochimie 95, 759–771 doi:10.1016/j.biochi.2012.11.003 23160073

[135] Zhang H., Chatterjee A. and Singh K.K. (2006) *Saccharomyces cerevisiae* polymerase ζ functions in mitochondria. Genetics 172, 2683–2688 doi:10.1534/genetics.105.051029 16452144 PMC1456388

[136] Kalifa L. and Sia E.A. (2007) Analysis of Rev1p and Pol ζ in mitochondrial mutagenesis suggests an alternative pathway of damage tolerance. DNA Repair 6, 1732–1739 doi:10.1016/j.dnarep.2007.06.005 17689152 PMC2129123

[137] Baruffini E., Serafini F., Ferrero I. and Lodi T. (2012) Overexpression of DNA polymerase zeta reduces the mitochondrial mutability caused by pathological mutations in DNA polymerase gamma in yeast. PLoS ONE 7, e34322 doi:10.1371/journal.pone.0034322 22470557 PMC3314619

[138] Lawrence C.W. and Maher V.M. (2001) Mutagenesis in eukaryotes dependent on DNA polymerase zeta and Rev1p. Philos. Trans. R. Soc. Lond. B Biol. Sci. 356, 41–46 doi:10.1098/rstb.2000.0001 11205328 PMC1087689

[139] Chatterjee N., Pabla R. and Siede W. (2013) Role of polymerase η in mitochondrial mutagenesis of *Saccharomyces cerevisiae*. Biochem. Biophys. Res. Commun. 431, 270–273 doi:10.1016/j.bbrc.2012.12.119 23313845

[140] Klingbeil M.M., Motyka S.A. and Englund P.T. (2002) Multiple mitochondrial DNA polymerases in *Trypanosoma brucei*. Mol. Cell 10, 175–186 doi:10.1016/S1097-2765(02)00571-3 12150917

[141] Seki M., Marini F. and Wood R.D. (2003) POLQ (Pol θ), a DNA polymerase and DNA-dependent ATPase in human cells. Nucleic Acids Res. 31, 6117–6126 doi:10.1093/nar/gkg814 14576298 PMC275456

[142] Seki M., Masutani C., Yang L.W., Schuffert A., Iwai S., Bahar I. et al. (2004) High-efficiency bypass of DNA damage by human DNA polymerase Q. EMBO J. 23, 4484–4494 doi:10.1038/sj.emboj.7600424 15496986 PMC526458

[143] Prasad R., Longley M.J., Sharief F.S., Hou E.W., Copeland W.C. and Wilson S.H. (2009) Human DNA polymerase θ possesses 5′-dRP lyase activity and functions in single-nucleotide base excision repair in vitro. Nucleic Acids Res. 37, 1868–1877 doi:10.1093/nar/gkp035 19188258 PMC2665223

[144] Black S.J., Kashkina E., Kent T. and Pomerantz R.T. (2016) DNA polymerase θ: a unique multifunctional end-joining machine. Genes 7, 67 doi:10.3390/genes7090067 PMC5042397 27657134

[145] Wisnovsky S., Jean S.R. and Kelley S.O. (2016) Mitochondrial DNA repair and replication proteins revealed by targeted chemical probes. Nat. Chem. Biol. 12, 567–573 doi:10.1038/nchembio.2102 27239789

[146] Harfe B.D. and Jinks-Robertson S. (2000) DNA polymerase ζ introduces multiple mutations when bypassing spontaneous DNA damage in *Saccharomyces cerevisiae*. Mol. Cell 6, 1491–1499 doi:10.1016/S1097-2765(00)00145-3 11163221

[147] Lawrence C.W. (2002) Cellular roles of DNA polymerase ζ and Rev1 protein. DNA Repair 1, 425–435 doi:10.1016/S1568-7864(02)00038-1 12509231

[148] Wachsmuth M., Hübner A., Li M., Madea B. and Stoneking M. (2016) Age-related and heteroplasmy-related variation in human mtDNA copy number. PLoS Genet. 12, e1005939 doi:10.1371/journal.pgen.1005939 26978189 PMC4792396

[149] Fuke S., Kubota-Sakashita M., Kasahara T., Shigeyoshi Y. and Kato T. (2011) Regional variation in mitochondrial DNA copy number in mouse brain. Biochim. Biophys. Acta, Bioenerget. 1807, 270–274 doi:10.1016/j.bbabio.2010.11.016 21145305

[150] Lin C.-S., Lee H.-T., Lee M.-H., Pan S.-C., Ke C.-Y., Chiu A.W.-H. et al. (2016) Role of mitochondrial DNA copy number alteration in human renal cell carcinoma. Int. J. Mol. Sci. 17, 814 doi:10.3390/ijms17060814 PMC4926348 27231905

[151] Wang Y., He S., Zhu X., Qiao W. and Zhang J. (2016) High copy number of mitochondrial DNA predicts poor prognosis in patients with advanced stage colon cancer. Int. J. Biol. Markers 31, e382–e388 doi:10.5301/jbm.5000211 27197581

[152] Reznik E., Miller M.L., Şenbabaoğlu Y., Riaz N., Sarungbam J., Tickoo S.K. et al. (2016) Mitochondrial DNA copy number variation across human cancers. eLife 5, e10769 doi:10.7554/eLife.10769 26901439 PMC4775221

[153] Vermulst M., Bielas J.H., Kujoth G.C., Ladiges W.C., Rabinovitch P.S., Prolla T.A. et al. (2007) Mitochondrial point mutations do not limit the natural lifespan of mice. Nat. Genet. 39, 540–543 doi:10.1038/ng1988 17334366

[154] Singh B., Li X., Owens K.M., Vanniarajan A., Liang P. and Singh K.K. (2015) Human REV3 DNA polymerase zeta localizes to mitochondria and protects the mitochondrial genome. PLoS ONE 10, e0140409 doi:10.1371/journal.pone.0140409 26462070 PMC4604079

[155] Maizels N. and Gray L.T. (2013) The G4 genome. PLoS Genet. 9, e1003468 doi:10.1371/journal.pgen.1003468 23637633 PMC3630100

[156] Bochman M.L., Paeschke K. and Zakian V.A. (2012) DNA secondary structures: stability and function of G-quadruplex structures. Nat. Rev. Genet. 13, 770–780 doi:10.1038/nrg3296 23032257 PMC3725559

[157] Zybailov B.L., Sherpa M.D., Glazko G.V., Raney K.D. and Glazko V.I. (2013) G4-quadruplexes and genome instability. Mol. Bio. 47, 197–204 doi:10.1134/S0026893313020180 23808155

[158] Dong D.W., Pereira F., Barrett S.P., Kolesar J.E., Cao K., Damas J. et al. (2014) Association of G-quadruplex forming sequences with human mtDNA deletion breakpoints. BMC Genomics 15, 677 doi:10.1186/1471-2164-15-677 25124333 PMC4153896

[159] Zybaĭlov B.L., Sherpa M.D., Glazko G.V., Raney K.D. and Glazko V.I. (2013) [G4-quadruplexes and genome instability]. Mol. Biol. (Mosk) 47, 224–231 23808155 10.7868/s0026898413020183

[160] Capra J.A., Paeschke K., Singh M. and Zakian V.A. (2010) G-quadruplex DNA sequences are evolutionarily conserved and associated with distinct genomic features in *Saccharomyces cerevisiae*. PLoS Comput. Biol. 6, e1000861 doi:10.1371/journal.pcbi.1000861 20676380 PMC2908698

[161] Oliveira P.H., Lobato da Silva C.L. and Cabral J.M.S. (2013) An appraisal of human mitochondrial DNA instability: new insights into the role of non-canonical DNA structures and sequence motifs. PLoS ONE 8, e59907 doi:10.1371/journal.pone.0059907 23555828 PMC3612095

[162] Bharti S.K., Sommers J.A., Zhou J., Kaplan D.L., Spelbrink J.N., Mergny J.-L. et al. (2014) DNA sequences proximal to human mitochondrial DNA deletion breakpoints prevalent in human disease form G-quadruplexes, a class of DNA structures inefficiently unwound by the mitochondrial replicative Twinkle helicase. J. Biol. Chem. 289, 29975–29993 doi:10.1074/jbc.M114.567073 25193669 PMC4208006

[163] Hixson J.E., Wong T.W. and Clayton D.A. (1986) Both the conserved stem-loop and divergent 5′-flanking sequences are required for initiation at the human mitochondrial origin of light-strand DNA replication. J. Biol. Chem. 261, 2384–2390 3944140

[164] Schiavone D., Jozwiakowski S.K., Romanello M., Guilbaud G., Guilliam T.A., Bailey L.J. et al. (2016) PrimPol is required for replicative tolerance of G quadruplexes in vertebrate cells. Mol. Cell 61, 161–169 doi:10.1016/j.molcel.2015.10.038 26626482 PMC4712188

[165] Paeschke K., Capra J.A. and Zakian V.A. (2011) DNA replication through G-quadruplex motifs is promoted by the *Saccharomyces cerevisiae* Pif1 DNA helicase. Cell 145, 678–691 doi:10.1016/j.cell.2011.04.015 21620135 PMC3129610

[166] Mislak A.C. and Anderson K.S. (2016) Insights into the molecular mechanism of polymerization and nucleoside reverse transcriptase inhibitor incorporation by human PrimPol. Antimicrob. Agents Chemother. 60, 561–569 doi:10.1128/AAC.02270-15 PMC4704227 26552983

[167] Sharma P.L., Nurpeisov V., Hernandez-Santiago B., Beltran T. and Schinazi R.F. (2004) Nucleoside inhibitors of human immunodeficiency virus type 1 reverse transcriptase. Curr. Top. Med. Chem. 4, 895–919 doi:10.2174/1568026043388484 15134548

[168] Vivet-Boudou V., Didierjean J., Isel C. and Marquet R. (2006) Nucleoside and nucleotide inhibitors of HIV-1 replication. Cell. Mol. Life Sci. 63, 163–186 doi:10.1007/s00018-005-5367-x 16389458 PMC11136153

[169] Reust C.E. (2011) Common adverse effects of antiretroviral therapy for HIV disease. Am. Fam. Physician 83, 1443–1451 21671545

[170] Arnaudo E., Dalakas M., Shanske S., Moraes C.T., DiMauro S. and Schon E.A. (1991) Depletion of muscle mitochondrial DNA in AIDS patients with zidovudine-induced myopathy. Lancet 337, 508–510 doi:10.1016/0140-6736(91)91294-5 1671889

[171] Peters B.S., Winer J., Landon D.N., Stotter A. and Pinching A.J. (1993) Mitochondrial myopathy associated with chronic zidovudine therapy in AIDS. Q. J. Med. 86, 5–15 8438050

[172] Lee H., Hanes J. and Johnson K.A. (2003) Toxicity of nucleoside analogues used to treat AIDS and the selectivity of the mitochondrial DNA polymerase. Biochemistry 42, 14711–14719 doi:10.1021/bi035596s 14674745 PMC7526745

[173] Feng J.Y., Johnson A.A., Johnson K.A. and Anderson K.S. (2001) Insights into the molecular mechanism of mitochondrial toxicity by AIDS drugs. J. Biol. Chem. 276, 23832–23837 doi:10.1074/jbc.M101156200 11328813

[174] Lim S.E. and Copeland W.C. (2001) Differential incorporation and removal of antiviral deoxynucleotides by human DNA polymerase γ. J. Biol. Chem. 276, 23616–23623 doi:10.1074/jbc.M101114200 11319228

[175] Johnson A.A., Ray A.S., Hanes J., Suo Z., Colacino J.M., Anderson K.S. et al. (2001) Toxicity of antiviral nucleoside analogs and the human mitochondrial DNA polymerase. J. Biol. Chem. 276, 40847–40857 doi:10.1074/jbc.M106743200 11526116

[176] Hanes J.W. and Johnson K.A. (2008) Exonuclease removal of dideoxycytidine (zalcitabine) by the human mitochondrial DNA polymerase. Antimicrob. Agents Chemother. 52, 253–258 doi:10.1128/AAC.00778-07 17984232 PMC2223897

[177] Guilliam T.A. and Doherty A.J. (2017) PrimPol — prime time to reprime. Genes 8, 20 doi:10.3390/genes8010020 PMC5295015 28067825

[178] Keen B.A., Bailey L.J., Jozwiakowski S.K. and Doherty A.J. (2014) Human PrimPol mutation associated with high myopia has a DNA replication defect. Nucleic Acids Res. 42, 12102–12111 doi:10.1093/nar/gku879 25262353 PMC4231748

[179] Gerhold J.M., Sedman T., Visacka K., Slezakova J., Tomaska L., Nosek J. et al. (2014) Replication intermediates of the linear mitochondrial DNA of Candida parapsilosis suggest a common recombination based mechanism for yeast mitochondria. J. Biol. Chem. 289, 22659–22670 doi:10.1074/jbc.M114.552828 24951592 PMC4132773

[180] Saxowsky T.T., Choudhary G., Klingbeil M.M. and Englund P.T. (2003) *Trypanosoma brucei* has two distinct mitochondrial DNA polymerase β enzymes. J. Biol. Chem. 278, 49095–49101 doi:10.1074/jbc.M308565200 12966090

[181] Hines J.C. and Ray D.S. (2010) A mitochondrial DNA primase is essential for cell growth and kinetoplast DNA replication in *Trypanosoma brucei*. Mol. Cell Biol. 30, 1319–1328 doi:10.1128/MCB.01231-09 20065037 PMC2832486

[182] Hines J.C. and Ray D.S. (2011) A second mitochondrial DNA primase is essential for cell growth and kinetoplast minicircle DNA replication in *Trypanosoma brucei*. Eukaryot. Cell 10, 445–454 doi:10.1128/EC.00308-10 21257796 PMC3067476

[183] Diray-Arce J., Liu B., Cupp J.D., Hunt T. and Nielsen B.L. (2013) The *Arabidopsis* At1g30680 gene encodes a homologue to the phage T7 gp4 protein that has both DNA primase and DNA helicase activities. BMC Plant Biol. 13, 36 doi:10.1186/1471-2229-13-36 23452619 PMC3610141

[184] Ono Y., Sakai A., Takechi K., Takio S., Takusagawa M. and Takano H. (2007) NtPolI-like1 and NtPolI-like2, bacterial DNA polymerase I homologs isolated from BY-2 cultured tobacco cells, encode DNA polymerases engaged in DNA replication in both plastids and mitochondria. Plant Cell Physiol. 48, 1679–1692 doi:10.1093/pcp/pcm140 17942449

[185] Backert S. and Börner T. (2000) Phage T4-like intermediates of DNA replication and recombination in the mitochondria of the higher plant *Chenopodium album* (L.). Curr. Genet. 37, 304–314 doi:10.1007/s002940050532 10853767

[186] Backert S., Dörfel P., Lurz R. and Börner T. (1996) Rolling-circle replication of mitochondrial DNA in the higher plant *Chenopodium album* (L.). Mol. Cell. Biol. 16, 6285–6294 doi:10.1128/MCB.16.11.6285 8887658 PMC231631

